# Effects of Methylprednisolone on Myocardial Function and Microcirculation in Post-resuscitation: A Rat Model

**DOI:** 10.3389/fcvm.2022.894004

**Published:** 2022-07-07

**Authors:** Changsheng Wang, Evelyne Bischof, Jing Xu, Qinyue Guo, Guanghui Zheng, Weiwei Ge, Juntao Hu, Elena Laura Georgescu Margarint, Jennifer L. Bradley, Mary Ann Peberdy, Joseph P. Ornato, Changqing Zhu, Wanchun Tang

**Affiliations:** ^1^Department of Emergency Medicine, Renji Hospital, Shanghai Jiao Tong University School of Medicine, Shanghai, China; ^2^Weil Institute of Emergency and Critical Care Research, Virginia Commonwealth University, Richmond, VA, United States; ^3^Department of Basic and Clinical Medicine, Shanghai University of Medicine and Health Sciences, Shanghai, China; ^4^Department of Medical Oncology, Renji Hospital, School of Medicine, Shanghai Jiao Tong University, Shanghai, China; ^5^Shanghai East International Medical Center, Shanghai, China; ^6^Department of Internal Medicine and Emergency Medicine, Virginia Commonwealth University Health System, Richmond, VA, United States; ^7^Department of Emergency Medicine, Virginia Commonwealth University Health System, Richmond, VA, United States

**Keywords:** cardiac arrest, methylprednisolone, microcirculation, myocardial dysfunction, inflammation

## Abstract

**Background:**

Previous studies have demonstrated that inflammation and impaired microcirculation are key factors in post-resuscitation syndromes. Here, we investigated whether methylprednisolone (MP) could improve myocardial function and microcirculation by suppressing the systemic inflammatory response following cardiopulmonary resuscitation (CPR) in a rat model of cardiac arrest (CA).

**Methods:**

Sprague-Dawley rats were randomly assigned to (1) sham, (2) control, and (3) drug groups. Ventricular fibrillation was induced and then followed by CPR. The rats were infused with either MP or vehicle at the start of CPR. Myocardial function and microcirculation were assessed at baseline and after the restoration of spontaneous circulation. Blood samples were drawn at baseline and 60-min post-resuscitation to assess serum cytokine (TNF-α, IL-1β, and IL-6) levels.

**Results:**

Myocardial function [estimated by the ejection fraction (EF), myocardial performance index (MPI), and cardiac output (CO)] improved post-ROSC in the MP group compared with those in the control group (*p* < 0.05). MP decreased the levels of the aforementioned pro-inflammatory cytokines and alleviated cerebral, sublingual, and intestinal microcirculation compared with the control (*p* < 0.05). A negative correlation emerged between the cytokine profile and microcirculatory blood flow.

**Conclusion:**

MP treatment reduced post-resuscitation myocardial dysfunction, inhibited pro-inflammatory cytokines, and improved microcirculation in the initial recovery phase in a CA and resuscitation animal model. Therefore, MP could be a potential clinical target for CA patients in the early phase after CPR to alleviate myocardial dysfunction and improve prognosis.

## Introduction

Cardiac arrest (CA) is a highly fatal medical emergency. Despite established guidelines, even an early intervention yields an approximately 50% success rate of resuscitation, of which only 0.8%-20.1% of patients reach a hospital discharge in the United States (1, 2). Myocardial dysfunction is the primary cause of early death in post-resuscitation (3–5), mostly caused by ischemia-reperfusion injury, stimulated inflammatory cascades, and elevated circulating catecholamines (6, 7). Microcirculatory blood flow is crucial for the recovery of vital organs following ischemia/reperfusion injury following CA (8). Our previous research revealed a close relationship between the severity of microcirculatory dysfunction and cardiopulmonary resuscitation (CPR) outcomes in a CA rat model (9).

Methylprednisolone (MP) is a non-halogenated corticosteroid with a methyl group at C6, which augments its anti-inflammatory properties. Several preclinical studies have found that MP attenuates macrophage and leukocyte activation, while decreasing the synthesis of inflammatory cytokines (10, 11). Endothelial damage is associated with systemic inflammation following myocardial injury (12). Post-resuscitation therapeutic strategies are aiming at to attenuating the inflammatory response following a successful CPR. Meanwhile, current research has indicated an enhanced survival rate in CA patients after administration of combined adrenaline, vasopressin, and MP (13). However, the effects of MP as a single drug therapy approach for improving cardiac function in CA patients post-CPR are not yet known. In this study, we investigated the cardioprotective effect of MP as a single agent in a post-resuscitation animal model.

## Materials and Methods

This work was approved by The Institutional Animal Care and Use Committee at the Virginia Commonwealth University and Guide for the Care and Use of Laboratory Animals (published by the National Institutes of Health) was followed while handling the rodents. All procedures were conducted in accordance with institutional guidelines. Envigo (a single-source breeder) was the source of 6–7-month-old healthy male Sprague-Dawley rats (weight range: 450–550 g) on which ventricular fibrillation was induced.

### Animal Preparation

Animals (*n* = 18) were fasting overnight, with access to water. Pentobarbital (45 mg/kg) was used to anesthetize the animals *via* intraperitoneal injection, with further administration of 10 mg/kg every hour if necessary to maintain the level of anesthesia. A 14-G cannula set on a blunt needle (Abbocath-T; Abbott Hospital Products Division, North Chicago, IL, United States) with a tip at 145 degrees was used for tracheal intubation. Constant monitoring of end-tidal CO_2_ (ETCO_2_) was performed using a side-stream infrared CO_2_ analyzer (Capstar-100; CWE Inc., PA or End-Til IL 200; Instrument Laboratory, Lexington, MA, United States) fitted between the ventilator and tracheal cannula. The pressure in the right atrium was quantified using a PE-50 catheter (Becton-Dickinson, Franklin Lakes, NJ, United States) with high-sensitivity transducers (model 42584-01; Abbott Critical Care Systems, North Chicago, IL, United States) placed into the right atrium through the left external jugular vein. A catheter was placed in the descending aorta through the left femoral artery. Blood temperature was quantified using a 10 cm long and 0.5 mm wide thermocouple microprobe (9030-12-D-34; Columbus Instruments, Columbus, OH) positioned into the left femoral vein.

To induce ventricular fibrillation (VF), a 3-F PE catheter (Model C-PMS-301J; Cook Critical Care, Bloomington, IN, United States) was inserted into the right atrium *via* the right external jugular vein. A saline solution containing 2.5 IU/mL of bovine crystalline heparin was introduced through the catheter regularly. To maintain the blood temperature (core temperature) at 37 ± 0.5°C, constant monitoring of a standard lead II ECG was used along with a heating blanket. The peritoneal cavity was exposed *via* laparotomy *via* a midline abdominal incision (∼1.5 cm). Dehydration and body heat loss were minimized by covering the wound with sterile gauze saturated with normal saline solution at 37°C. A craniotomy was performed, and the right parietal bone was excised without damaging the dura mater (14).

### Experimental Procedures and Treatments

The rats were randomly assigned (by a simple randomization method, where the degree of freedom (E) of variance analysis is E = number of all animals-the number of groups) to one of three groups (six animals per group): (1) drug: rats in this group underwent VF, CPR, and received MP treatment; (2) control: rats in this group underwent VF, CPR, and received saline; and (3) sham: animal preparation was the same but without VF induction or CPR.

A baseline measurement was performed 15 min before VF induction, while mechanical ventilation was initiated at 100 breaths per min (frequency), using a volume of 0.60 ml/100 g of body weight (tidal volume) with an inspired O_2_ fraction (FiO_2_) of 0.21. Electrical induction of VF involved a cumulative current administered to the endocardium of the right ventricle with a 60-Hz voltage and a maximum of 3.5 mA intensity current.

Spontaneous defibrillation was prevented by continuing the current flow for 3 min. Mechanical ventilation was stopped at the start of VF. Initiation of precordial compression (PC) was done 8 min after the starting of VF and was coupled with mechanical ventilation at a frequency of 100 breaths a minute with FiO_2_ of 1.0. The PC was kept constant at 200/min and was coordinated with the number of ventilations in order to supply a 2:1 compression/ventilation ratio. The compression and relaxation were equal. The CPP (coronary perfusion pressure) was maintained at 22 ± 2 mmHg while the ETCO_2_ was 11 ± 2 mmHg after an initial adjustment of the compression depth. Resuscitation consisted of up to three four-joule counter shocks. CPR (30s intervals) was followed by a subsequent sequence of up to three shocks in the case of failure of ROSC (return of spontaneous circulation). One minute after PC initiation, an injection of 30 mg/kg of MP (8% concentration diluted in 0.375 ml/kg NaCl 0.9%) or 0.375 ml/kg vehicle (saline 0.9%) into the right atrium was performed over a 10-sec interval. ROSC was considered the maintenance of an average aortic pressure over 50 mmHg for at least 5 min. Once ROSC was achieved, mechanical ventilation was continued using 100% oxygen for the first 60 min, 50% for the next 60 min and 21% afterward. Euthanization was performed by administrating an overdosage of 150 mg/kg pentobarbital *via* the femoral artery. The injuries induced by the surgery to the viscera, thorax, and abdominal vessels were assessed during necropsy. Supplementary Figure 1 illustrates the experimental protocol of the study.

### Quantifying Hemodynamics

A personal computer-based data acquisition system (DATAQ Instruments, Akron, OH) was used to record the pressures inside the aorta and the right atrium, the electrocardiogram, and the ETCO_2_ for a period of 6 h. The CPP was calculated as the difference in real-time between pressures.

### Echocardiography Measurements

Myocardial function was quantified by evaluating the ejection fraction (EF), myocardial performance index (MPI), and cardiac output (CO) at baseline and 2, 4, and 6 h post-ROSC using echocardiography (HD 11 XE, Philips Healthcare, Andover, MA, United States) with a 12.5 MHz transducer (15). Two investigators performed the quantifications individually and were blinded to the study groups.

### Microcirculation Evaluation

Cerebral, sublingual, and intestinal microcirculation was monitored at baseline and 1, 3, and 6 h post-ROSC using a 5 × optical probe containing side stream dark-field imaging (MicroScan; MicroVision Medical Inc., Amsterdam, Netherlands). A sublingual microcirculation assessment was performed at the base of the tongue. The approach of Qian et al. was applied to measure intestinal microcirculation (16). Brain microcirculation was observed in the right parietal lobe. The microcirculatory flow index (MFI) assessed the blood flow in vessels with a diameter of less than 20 μm. Vascular density was quantified using images, as described by De Backer et al. (17). Functional capillary density was estimated using the perfused vessel density (PVD) parameter calculated as the product of the perfused vessel proportion and vessel density. The average values for the three areas were calculated for each animal.

### Enzyme-Linked Immunosorbent Assay

To quantify TNF-α, IL-6, and IL-1β serum cytokine levels, blood samples (1 ml) were collected at baseline and 1h after ROSC, followed by immediate centrifugation at 4000 rpm at room temperature for 10 min and stored until analysis. An enzyme-linked immunosorbent assay (ELISA) (R&D Systems, Minneapolis, MN) was used to quantify the cytokines in accordance with the manufacturer’s protocol.

### Statistical Analysis

Data are presented as mean ± SD or median (interquartile range). All variables were compared using analysis of variance (ANOVA) (parametric test) or the Mann-Whitney U test (non-parametric test). Repeated-measurement analysis of variance was conducted for intragroup alterations of time-based measurements. After obtaining a difference between the groups, we used the Bonferroni test to compare any other two groups. Pearson’s correlation coefficient was calculated for linear correlations. Statistical significance was set at a *p*-value of < 0.05.

## Results

All 18 rats were successfully resuscitated. The electrical current required to induce VF and CPP during CPR and the number of defibrillations necessary to restore ROSC were comparable among all groups. CPP was 22 ± 2.2 mm Hg and 23.5 ± 2.4 mm Hg in the drug and control group, respectively. The number of defibrillations were 1.3 ± 0.5 and 1.9 ± 1.6 in the drug and control group, respectively.

### Effect of Methylprednisolone on Post-resuscitation Hemodynamics

**FIGURE 1:**
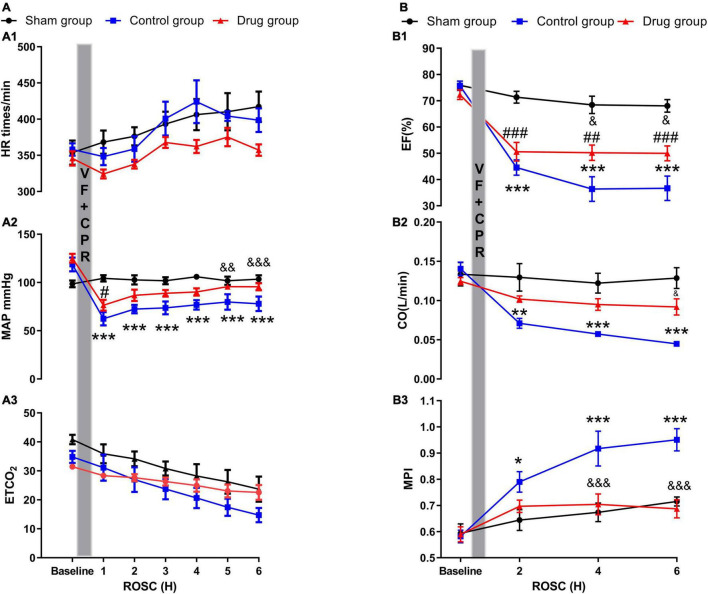
Myocardial performance and myocardial function. **(A1)** Heart rate; **(A2)** mean arterial pressure; **(A3)** end-tidal CO_2_; **(B1)** ejection fraction; **(B2)** cardiac output; **(B3)** myocardial performance index. HR, heart rate; MAP, mean arterial pressure; ETCO_2_, end-tidal CO_2_; CO, cardiac output; EF, ejection fraction; MPI, myocardial performance index; VF, ventricular fibrillation; CPR, cardiopulmonary resuscitation; ROSC, return of spontaneous circulation. Values are presented as mean ± SD (*n* = 6 in each group). *p < 0.05, **p < 0.01, ***p < 0.001 control vs. sham group; ^&^*p* < 0.05, ^&⁣&^*p* < 0.01, ^&⁣&⁣&^*p* < 0.001 drug vs. control group; ^#^*p* < 0.05, ^##^*p* < 0.01, ^###^*p* < 0.001, drug vs. sham group.

No evident differences between baseline hemodynamics were observed (Supplementary Table 1). Post-resuscitation, there were no differences in heart rate and ETCO_2_ between the animals in the drug and control groups. The drug and control groups displayed a reduction in mean arterial pressure (MAP) following resuscitation compared to the baseline and sham groups. However, MP administration caused a significant increase of MAP 5 and 6 h post-ROSC, which was not observed in the control group (Figure 1).

### Effect of Methylprednisolone on Myocardial Function Post-resuscitation

The three groups displayed no differences in myocardial function (EF, CO, and MPI) at baseline (Supplementary Table 1). There was an evident reduction in all three functions post-resuscitation compared with their baselines in both the drug and control group. However, in MP-treated animals, the severity of the myocardial dysfunction post-resuscitation, especially MPI, was significantly lower compared with control animals at the time points of 2, 4, and 6 h of ROSC (Figure 1).

### Methylprednisolone Attenuated System Inflammation

**FIGURE 2:**
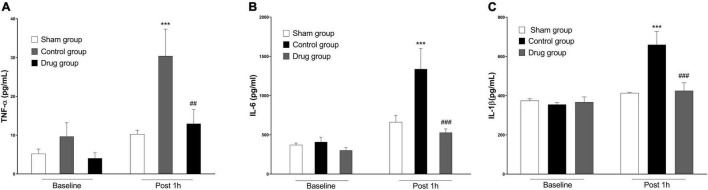
MP effects on post-resuscitation pro-inflammatory cytokines. **(A)** Plasma levels of TNF-α, **(B)** plasma levels of IL-6, **(C)** plasma levels of IL-1β. TNF-α, tumor necrosis factor-α; IL-6, interleukin 6; IL-1β, interleukin 1β. Data are presented as mean ± SD. ***p < 0.001 control vs. sham group; ^##^*p* < 0.01, ^###^*p* < 0.001, drug vs. control group.

Serum cytokine levels were increased in post-resuscitation state in both drug and control groups compared with sham. However, TNF-α, IL-1β, and IL-6 levels were significantly lower in animals treated with MP at 1 h post-resuscitation as compared to the control animals (Figure 2).

### Effect of Methylprednisolone on Post-resuscitation Microcirculation

**FIGURE 3:**
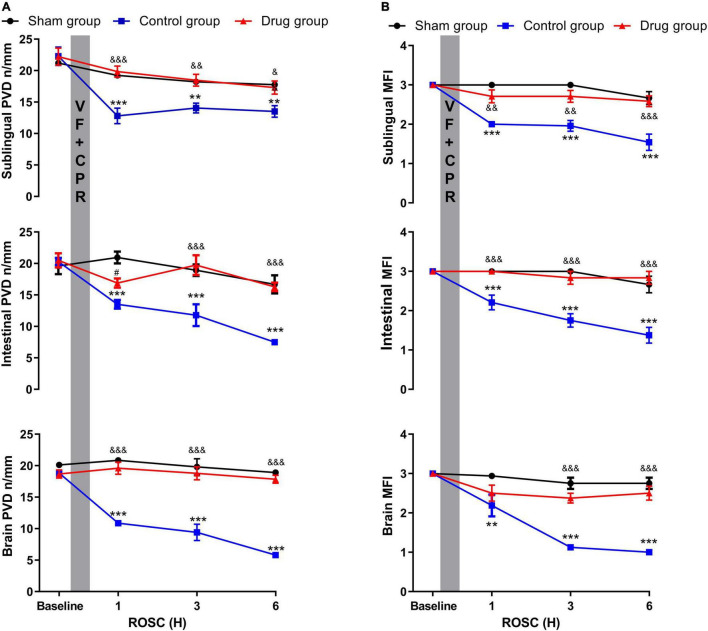
MP effects on post-resuscitation microcirculation. **(A)** Perfused vessel density, **(B)** microcirculatory flow index. PVD, perfused vessel density; MFI, microcirculatory flow index; VF, ventricular fibrillation; CPR, cardiopulmonary resuscitation. Data were presented as mean ± SD (*n* = 6 in each group). **p < 0.01, ***p < 0.001 control vs. sham group; ^&^*p* < 0.05, ^&⁣&^*p* < 0.01, ^&⁣&⁣&^*p* < 0.001, drug vs. control group; ^#^*p* < 0.05, drug vs. sham group.

Decrease in cerebral, intestinal, and sublingual PVD and MFI were observed at 1, 3, and 6 h post-resuscitation in control animals. However, in MP-treated animals, this reduction was reduced to a non-detectable level, which was not observed in the baseline and sham groups (Figure 3 and Supplementary Figure 2). Microcirculatory blood flow was negatively correlated with inflammatory cytokine levels (Table 1).

**TABLE 1:** Correlations between microcirculatory parameters and serum cytokine levels.

	Sublingual		Cerebral		Intestinal	
	PVD	MFI	PVD	MFI	PVD	MFI

**Cytokine levels**	***r***	***r***	***r***	***r***	***r***	***r***
TNF-α	−0.425*	−0.752*	−0.434^#^	−0.608*	−0.461*	−0.651*
IL-1β	−0.553*	−0.819*	–0.310	−0.467^#^	−0.480*	−0.644*
IL-6	−0.630*	−0.699*	−0.532*	−0.621*	−0.498*	−0.801*

PVD, perfused vessel density; MFI, microcirculatory flow index; TNF-α, tumor necrosis factor α; IL-6, Interleukin-6; IL-1β, Interleukin-1β. ^#^p < 0.05, *p < 0.01.

## Discussion

In our study, we observed that the administration of MP improved the objective myocardial function in post-resuscitation model animals who suffered from a global ischemia caused by a CA. Reduction of inflammation and improvement of microcirculation-induced ischemia following CPR are plausible reasons for this outcome.

Several clinical studies indicated that high post-resuscitation mortality rates are linked to substantial myocardial dysfunction following successful resuscitation from CA (4, 5). Similarly, in the present study, the initial post-resuscitation myocardial function was strongly impaired. However, the severity of the dysfunction was significantly reduced in MP-treated rats. In addition to improved myocardial function, we observed a reduction in systemic inflammation and an improvement in microcirculatory blood flow. Clinically, the robust anti-inflammatory effect of MP allows for its use in diseases characterized by inflammation, such as ulcerative colitis, Crohn’s disease, and rheumatoid arthritis. In addition, pro-inflammatory cytokines TNF-α and IL-1β engage in the pathogenesis of myocardial dysfunction and cardiomyocyte death in I/R injury has been described (18). Adrie et al. showed that elevated levels of circulating cytokines in the plasma, along with the aberrant cytokine balance found in CPR patients is similar to the immunological disturbances found in patients with sepsis (19). Furthermore, a recent multicenter observational study found that higher levels of IL-1Ra, IL-6, IL-8, and IL-10 were present in non-survivors and survivors with poor functional outcomes (20). Moreover, an elevated IL-6 level following ROSC has been linked to worse outcomes (21, 22). In the present study, we demonstrated that systemic inflammatory cytokines, such as TNF-α, IL-6 and IL-1β measured in the post-resuscitation state, were significantly reduced by MP in a rat model of CA post-CPR. This result indicates that the reduction in pro-inflammatory cytokines caused by MP might be a possible mechanism for the increased post-resuscitation myocardial function observed in our study.

We further demonstrated that MP improved post-resuscitation microcirculation. Microcirculatory dysfunction was minimal in MP-treated animals, with the baseline value being almost reached at 1 h post-ROSC and maintained during the 6-h observation post-resuscitation period. In MP-treated animals, an increase in microcirculatory blood flow was negatively correlated with a decrease in inflammatory cytokine levels. The close association between the severity of microcirculatory dysfunction and vital organ function post-resuscitation has received significant attention among cardiology and intensive medicine science (16, 23). Our previous work in animals also showed that microcirculatory flow and serum cytokine levels are closely linked (9, 16). Improvement in microcirculation after myocardial ischemia/reperfusion in STZ-induced diabetic animals treated with MP *via* TLR4/NF-κB signaling inhibition has been reported (24). We also found that administration of MP after CPR increased the MAP. Increased post-resuscitation microcirculation indicates enhanced organ perfusion. Overall, our results show that post-resuscitation “sepsis-like” syndrome triggered by whole-body I/R is most likely the underlying mechanism of microcirculatory dysfunction after successful resuscitation. Moreover, microcirculatory and myocardial functions can be increased in the post-resuscitation state by administration of anti-inflammatory drugs.

Although there are clinical trials that show that hospitalized CA patients receiving vasopressin plus methylprednisolone or epinephrine at resuscitation do not have an improved 30-day survival, several randomized, double-blind trials, published in 2009 and 2013, Mentzelopoulos et al. (25, 26) and Andersen et al. (27) compared the addition of vasopressin and glucocorticoids during CA with placebo, showing a large improvement in outcomes. Moreover, in this study, we have implemented a ventricular fibrillation animal model.

Since a single dose of MP was used in the study, further protocols with varying MP administration patterns for effect comparison are pending. Furthermore, a limitation occurred since the study focused on measuring of the pro-inflammatory cytokines at 1 h post-resuscitation, there are no conclusions to be drawn regarding their changes in further post-resuscitation recovery. Additionally, intergroup survival analyses were not performed.

Another limitation is of methodological nature: MFI is based on a semiquantitative scoring system to assess the magnitude of microvascular perfusion and is thus considered to be a subjective parameter and was not recommended in the second consensus. Furthermore, the Microvision camera we have used is designed for analyzing sublingual microcirculation. While data on any other microvascular beds are limited, several studies have investigated intestinal and brain microcirculation by the Sidestream Dark-Field (SDF) imaging method (28, 29). This technique has been found as highly promising for the visualization and assessment of bowel microcirculation (30) in a study performed on patients during gastrointestinal surgery.

Sidestream dark field imaging is a very promising technique for bowel microcirculatory visualization and assessment.

In conclusion, the present study showed that MP reduced the severity of post-resuscitation myocardial dysfunction by targeting pro-inflammatory cytokines and improving microcirculation. Therefore, our study suggests that MP could be a potential therapeutic for ROSC patients after a CA, especially when administered at an early phase after CPR, in order to alleviate myocardial dysfunction and improve prognosis.

## Data Availability Statement

The raw data supporting the conclusions of this article will be made available by the authors, without undue reservation.

## Ethics Statement

This work was approved by the Institutional Animal Care and Use Committee at the Virginia Commonwealth University and Guide for the Care and Use of Laboratory Animals.

## Author Contributions

CW, EB, JX, QG, GZ, WG, JH, and JB: data collection, statistical analysis, data analysis and interpretation, manuscript preparation. CW, MP, JO, CZ, and WT: conception and design of the study, data analysis, and interpretation. EG: visualization and review. CZ and WT: senior reviewer. All authors have read and approved the manuscript.

## Conflict of Interest

The authors declare that the research was conducted in the absence of any commercial or financial relationships that could be construed as a potential conflict of interest.

## Publisher’s Note

All claims expressed in this article are solely those of the authors and do not necessarily represent those of their affiliated organizations, or those of the publisher, the editors and the reviewers. Any product that may be evaluated in this article, or claim that may be made by its manufacturer, is not guaranteed or endorsed by the publisher.
