# Supplementary material for: Effects of Methylprednisolone on Myocardial Function and Microcirculation in Post-resuscitation: A Rat Model
Source: Front Cardiovasc Med. 2022 Jul 7;9:894004. doi: 10.3389/fcvm.2022.894004 (PMC9301050; doi:10.3389/fcvm.2022.894004)
Supplement: Supplementary file 1 [file Data_Sheet_1.docx]

Supplementary Material

# Supplementary Figures and Tables

## Supplementary Figures


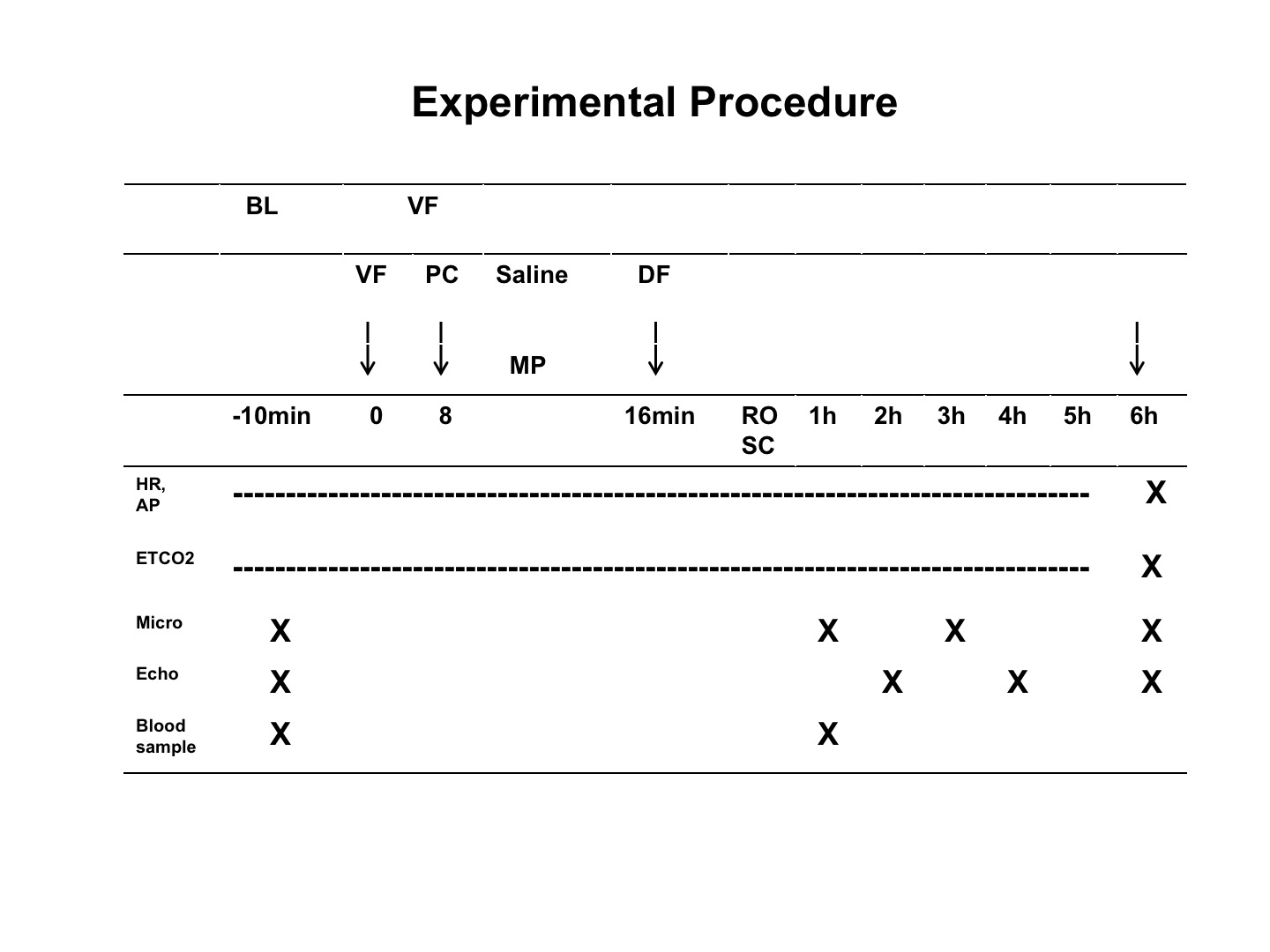


**Supplementary Figure 1.** Experimental outline and procedure. BL, baseline; VF, ventricular fibrillation; PC, precordial compression; MP, methylprednisolone; DF, defibrillation; ROSC, return of spontaneous circulation; HR, heart rate; AP, arterial pressure; ETCO2, end-tidal CO2; Micro, microcirculation; Echo, echocardiography.


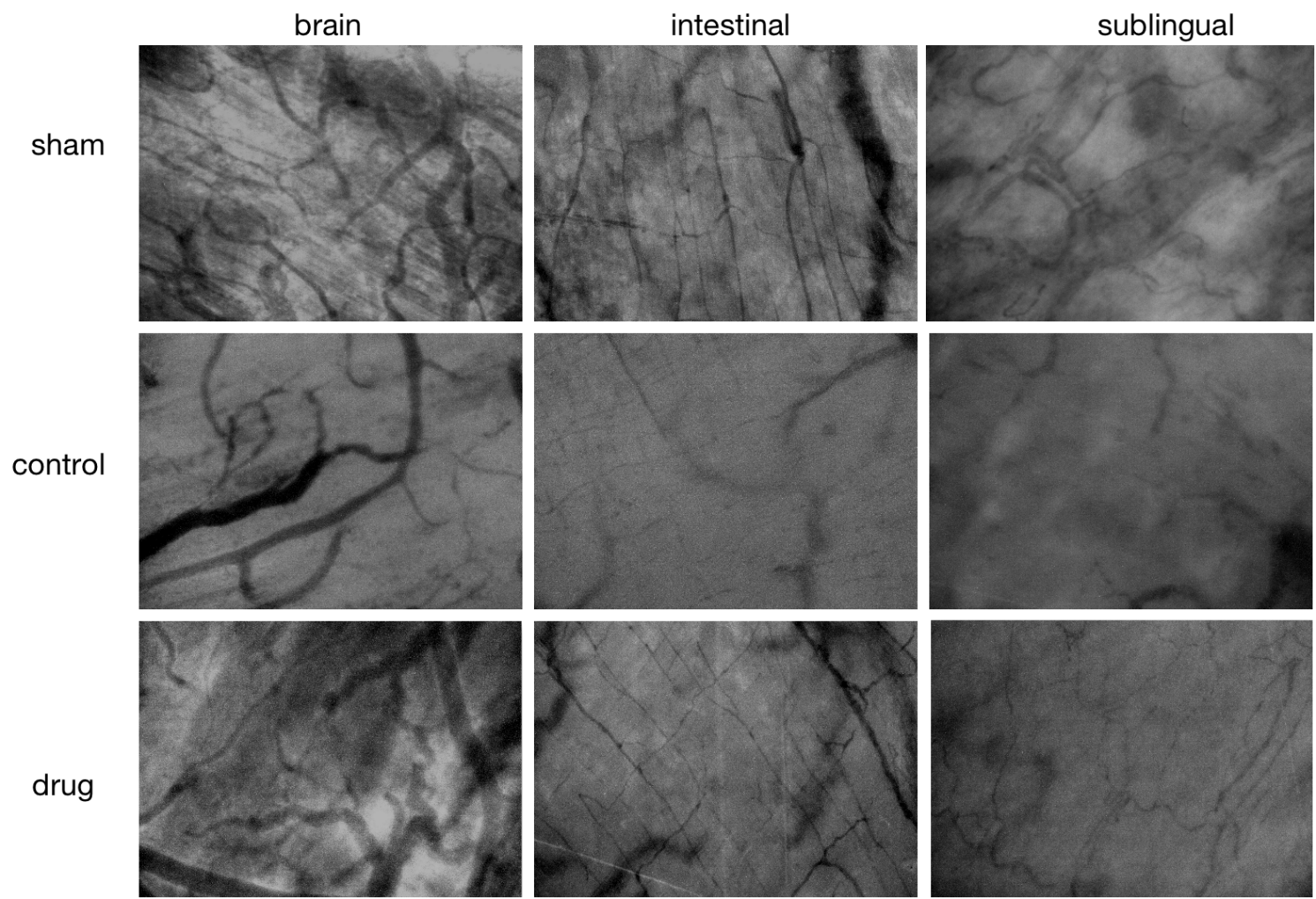


**Supplementary Figure 2.** Images of brain, sublingual, and intestinal microcirculation obtained by the sidestream dark-field (SDF) video microscope at 6 h post-resuscitation of the three groups studied.

## Supplementary Table 1. Baseline characteristics of the three groups evaluated.

| Group | Sham | Control | MP |
| --- | --- | --- | --- |
| Body weight, g | 509.4±6.3 | 502.6±11.8 | 506.6±10.4 |
| Heart rate, bpm | 354±41 | 358±21 | 346±24 |
| MAP, mmHg | 98±9 | 118±17 | 125±10 |
| End-tidal CO2, mmHg | 31.4±2.2 | 34.8±5.1 | 40.8±4.0 |
| Ejection fraction, % | 75.9±3.9 | 75.7±4.4 | 72.3±4.5 |
| Cardiac output, mL/min | 133±3.7 | 140±2.1 | 124±1.1 |
| MPI | 0.59±0.08 | 0.58±0.05 | 0.58±0.07 |

MP, methylprednisolone; MAP, mean aortic pressure; MPI, myocardial performance index. Values are presented as mean±SD. No significant differences were found between the groups.
